# Design considerations of a randomized clinical trial on a cognitive behavioural intervention using communication and information technologies for managing chronic low back pain

**DOI:** 10.1186/1471-2474-14-142

**Published:** 2013-04-22

**Authors:** Julio Domenech, Rosa Baños, Lourdes Peñalver, Azucena Garcia-Palacios, Rocio Herrero, Aida Ezzedine, Monica Martinez-Diaz, Javier Ballester, Jaime Horta, Cristina Botella

**Affiliations:** 1 Orthopaedic Surgery Department, Hospital Arnau de Vilanova, C/ San Clemente, 46015, Valencia, Spain; 2 Faculty of Health Sciences, University Cardenal Herrera CEU, Valencia, Spain; 3 Department of Personality, Evaluation and Psychological Treatments, Faculty of Psychology, University of Valencia, Valencia, Spain; 4 Department of Physical Therapy and Rehabilitation, Hospital Arnau de Vilanova, Valencia, Spain; 5 Department of Clinical and Basic Psychology and Biopsychology, Faculty of Psychology, University Jaume I, Castellon, Spain

**Keywords:** Low back pain, Cognitive behavioural therapy, Physical therapy, Information and communication technologies

## Abstract

**Background:**

Psychological treatments have been successful in treating chronic low back pain (CLBP). However, the effect sizes are still modest and there is room for improvement. A way to progress is by enhancing treatment adherence and self-management using information and communication technologies (ICTs). Therefore, the objective of this study was to design a trial investigating the short- and long-term efficacy of cognitive behavioural treatment (CBT) for CLBP using or not ICTs. A secondary objective of this trial will be to evaluate the influence of relevant variables on treatment response. Possible barriers in the implementation of CBT with and without ICT will also be investigated.

**Methods:**

A randomised controlled trial with 180 CLBP patients recruited from specialised care will be conducted. Participants will be randomly assigned to three conditions: Control group (CG), CBT, and CBT supported by ICTs (CBT + ICT). Participants belonging to the three conditions will receive a conventional rehabilitation program (back school). The CBT group program will last six sessions. The CBT + ICT group will use the internet and SMS to practice the therapeutic strategies between sessions and in the follow-ups at their homes. Primary outcome variables will be self-reported disability and pain intensity. Assessment will be carried out by blinded assessors in five moments: pre-treatment, post-treatment and 3-, 6-, and 12-month follow-ups. The influence of catastrophizing, fear-avoidance beliefs, anxiety and depression in response to treatment in the primary outcomes will also be analysed.

**Discussion:**

This study will show data of the possible benefits of using ICTs in the improvement of CBT for treating CLBP.

**Trial registration:**

ClinicalTrials.gov, NCT01802671

## Background

Low back pain is a prevalent health condition and a leading cause of disability. It is associated with growing healthcare costs in developed countries, affecting 70% of the general population at some point in their lives, with an annual incidence of 40% [1]. Low back pain presents a tendency to become chronic or produce relapses that can severely impair daily lives of sufferers. In a survey carried out in a Spanish sample, 69% of participants reported low back pain lasting more than three years [2]. Chronic low back pain (CLBP) is the most frequent cause of sick leave and the leading cause of disability in people less than 45 years. The economic burden is approximately 1.7% of the gross domestic product in developed countries and it has been suggested that this problem is even worse in Spain [3]. In this country, CLBP causes 54.8% of days off work [4]. This problem is acquiring epidemic dimensions. In Spain, the number of low back pain episodes has increased from 43,328 in 1993 to 122,995 in 2004, an increase of 183.8% [5].

In 85% of cases, it is not possible to find a precise cause of the pain [1]. The correlation between the symptoms and the MRI findings is very low and there is no relationship between the tendency to chronicity and structural alterations of the spine [6-8]. The patients’ beliefs and attitudes, mainly those related to fear and avoidance, are powerful predictors of CLBP [9-12].

Conservative and interventional medical treatments have a low to moderate effect in CLBP [13]. Empirical evidence suggests that structural changes have a low impact in the treatment of CLBP [7]. Catastrophizing and fear-avoidance beliefs seem to counteract the beneficial effects of conventional and rehabilitation treatment [14,15]. Biopsychosocial perspectives propose taking into consideration structural alterations, as well as psychological and social factors. Although clinical guidelines recommend avoiding rest, promoting activity despite pain and an early return to work, these recommendations have low impact on the improvement of patients. A systematic review about the effect of written information on patients indicated that there was limited evidence of the superiority of a biopsychosocial information brochure compared to a biomedical one on modifying patients’ beliefs about physical activity. Moreover, this approach did not produce changes in pain and disability [16].

Psychological treatments go beyond information about pain. Cognitive behavioural interventions (CBT) address dysfunctional behaviours and beliefs to change behavioural and thinking patterns. These interventions have been successful in treating chronic pain, including CLBP [17]. CBT elicits clinical improvement similar to that achieved with lumbar fusion [18,19]. The COST B13 European guideline [13] recommends CBT in patients with CLBP, especially when surgical interventions are being considered.

Despite the promising results of CBT, it is important to highlight the fact that effect sizes are still modest and that most studies do not include long-term follow-ups. It is necessary to improve CBT programs for CLBP to obtain better therapeutic outcomes and for longer terms. One possible way of improving these programs is by promoting the review of the therapy session contents outside the clinic during the therapy program and once the program is finished. This can be achieved by using information and communication technologies (ICTs) to enhance treatment adherence and self-management.

ICTs have been used already in pain management. There are several studies supporting the efficacy of virtual reality (VR) as a powerful distraction technique for acute pain control associated with medical procedures (see Malloy and Milling, 2010 [20] and Keefe et al., 2012 [21] for a review). There are some preliminary data supporting the use of VR as an adjunct to CBT in fibromyalgia [22] and other medical conditions such as cancer [23]. Another ICT that has been used is the internet; it is now well established in dozens of randomised controlled trials that internet-based therapies are effective in treating several problems [24,25]. Our research group has contributed to this line of research in the field of online therapy for anxiety disorders. In 2000, we developed *“Talk to Me”* and *“Without Fear”*, two internet-based self-applied programs for treating social phobia and small animal phobias [26]. Several case studies and two controlled investigations have shown the efficacy of these programs [27,28]. In the field of chronic pain, there is some evidence of the utility of this ICT in the treatment of several conditions like headache, rheumatoid arthritis, osteoarthritis and fibromyalgia [29-32].

Internet-based interventions are very attractive because they address an important issue in the management of chronic diseases: the possibility of systematic training in self-management strategies. Patients learn and practice therapeutic strategies in their natural environment and on their own, promoting self-management and self-efficacy. In fact, a meta-analysis comparing online and traditional interventions reported a higher increase in knowledge and positive behavioural changes in participants with chronic conditions who had participated in an online program [33].

We have developed an internet-based intervention program called TEO (Terapia Emocional Online) that includes two platforms (therapist and patient platform). The system allows the development and management of different therapeutic contents using multimedia elements (therapeutic information and exercises). Patients and therapists can access these contents online. The aim of this application is to practice the therapeutic strategies outside the consultation room as homework and also during the follow-up periods after the treatment programs have finished.

The objective of this study was to design a randomised controlled trial that investigates the short- and long-term efficacy of a CBT program using ICT or not in CLBP treatment. A secondary objective of this trial will be to evaluate the effect of dysfunctional attitudes and beliefs (catastrophizing and fear-avoidance beliefs), depression and anxiety on treatment response. Possible barriers in the implementation of CBT with and without ICT will also be investigated.

## Methods/design

### Study design

The study will consist of a three-armed (Control, CBT and CBT+ICT) simple-blind randomised controlled trial, with five assessment periods (pre-treatment, post-treatment, and three follow-ups at 3, 6 and 12 months). The study will follow the CONSORT guideline [34].

### Study population

The clinical trial will be conducted in the Valencia-Llíria Health Department in Valencia, Spain. This attends to a population of 320,000 inhabitants. The participants will be referred by medical doctors from the Orthopaedics and Rehabilitation Units. One hundred and eighty participants with a diagnosis of non-specific chronic low back pain according to the definition established by the COST B-13 guideline will be included in the investigation. Patients consecutively referred from primary and specialised care will be invited to participate. Written informed consent will be obtained from all participants. The inclusion criteria are: age between 20 and 65 years; low back pain for at least 6 months; availability of a mobile phone to receive SMS; access to a computer with internet connection to be able to use the CBT program supported by ICT. Exclusion criteria are: mental retardation; not proficient in Spanish; neurogenic claudication or neurologic deficit; history of vertebral fracture; previous lumbar surgery; vertebral infection; spinal or nerve tumour; severe mental disorder; or substance abuse or dependence.

### Randomization

Participants will be randomly assigned to one of three conditions (Control, CBT and CBT +ICT). Participants will be stratified according to their level of disability: severe (Roland Morris Disability Questionnaire (RMDQ) score > 15), moderate (RMDQ score between 8 and 14) and mild (RMDQ score <7). Allocation will be performed by a computer-generated list according to the degree of disability at baseline. It will be supervised by a statistician not belonging to the research group and unaware of the study characteristics.

During recruitment, the allocation sequence will be concealed. Due to the nature of the interventions, it is not possible to blind the participants. The physiotherapists administering the sessions and the assessors will be unaware of the participants’ allocation. Data analysis will be conducted without being aware of the intervention received by the participants.

### Sample size

The study has been designed taking into consideration changes of 2 points in the pain intensity measured by the numerical rating scale (NRS) and of 3 points in the RMDS. These changes have been recognised as clinically relevant [35]. According to the results of a previous CLBP sample treated in our health centre, the mean disability according to the RMDS presented a standard deviation of 4, while the mean pain intensity displayed a standard deviation of 2. Considering these data and an alpha of 0.01 and a beta of 0.9 for two-tailed mean tests, the required sample size is 53 to detect changes in disability and 30 to detect changes in pain intensity. Considering an attrition of 15%, the sample will need 60 participants per condition. For further analysis of treatment response according to high or low catastrophizing, fear-avoidance beliefs, depression and anxiety, disability measured by the RMDS will be considered with an alpha of 0.05 and beta of 0.8, resulting in a sample size of 28 participants per group.

### Outcome measures

#### Primary outcomes

**Disability** The Roland-Morris (RM) questionnaire is one of the most widely used measures to assess disability in patients with low back pain [36]. This questionnaire was designed to evaluate low back pain, with 24 statements that describe different daily activities that can be affected by low back pain. The participant has to select the statements that describe the limitations produced by their low back pain. The Spanish version of the RM has shown good psychometric properties (reliability and validity) [37].

**Pain** IMMPACT recommends the use of NRS as a core outcome measure of efficacy in clinical trials of chronic pain treatments [38]. The scale is composed of 11 numbers ranging from 0 to 10, with 0 meaning ‘No pain’ and ‘10’ meaning ‘Pain as bad as you can imagine’. Two different scales will be used, one to assess low back pain and the other to examine sciatica pain.

#### Secondary outcomes

**Pain coping strategies** The Coping Strategies Questionnaire (CSQ) assesses the frequency of several cognitive and behavioural strategies to cope with pain [39]. Patients must select in a Likert-type scale how often they use each strategy. It comprises seven subscales, six for cognitive strategies (ignoring pain, reinterpretation of pain, diverting attention, coping self-statements, catastrophizing and praying/hoping) and one for behavioural strategies (activity level). This questionnaire has been validated in the Spanish population and has shown good psychometric properties for evaluating patients with chronic musculoskeletal pain [40].

**Anxiety and depression** Levels of anxiety and depression will be assessed using the Hospital Anxiety and Depression Score (HADS) questionnaire. This questionnaire is widely used in clinical practice [41]. It is a fourteen-item scale where seven of the items are related to anxiety and the other seven to depression. The Spanish version has shown good internal consistency and external validity, as well as an adequate sensitivity to identify clinically significant depression [42].

**Fear-avoidance beliefs** The Fear-Avoidance Beliefs Questionnaire (FABQ) will be used to measure this domain. Is a scale composed of sixteen items developed to assess patients’ beliefs and attitudes about the causes and consequences of their low back pain [43]. Participants rate their agreement with each statement on a 7-point Likert scale (0 = completely disagree, 6 = completely agree). The FABQ consists of 2 subscales. The first sub-scale is the Physical Activity subscale (FABQpa), composed of seven items, which assesses the effects of physical activities on pain. The second subscale is the Work subscale (FABQw), composed of four items, which assesses the way that work activities can affect pain. The Spanish version has shown excellent psychometric properties [44].

**Catastrophizing** The Pain Catastrophizing Scale (PCS) tests the tendency to consider pain as a threat with exaggerated negative consequences in a patient [45]. It is a 13-item self-report scale, with each item evaluating the pain experience. For example: “I become afraid that the pain will get worse”. Participants assess the frequency at which these ideas appear on a Likert-type scale ranging from 0 to 4, with 0 being “not at all” and 4 “all the time”. The PCS yields a total score and three subscale scores examining rumination, magnification and helplessness. A high score on the total PCS score indicates a high level of catastrophizing. The Spanish version of PCS has demonstrated adequate psychometric properties [46].

**Quality of life** The SF-12 is the brief version of the 36-item Short Form Health Survey (SF-36). It contains items that measure each of the eight concepts included in the SF-36, namely physical functioning, role limitations due to physical health problems, bodily pain, general health, vitality (energy/fatigue), social functioning, role limitations due to emotional problems and mental health (psychological distress and psychological well-being). The SF-12 contains six-point scales in which patients evaluate the frequency and intensity of each statement over the past month [47,48].

#### Other measures

Demographic information will be collected on the following: age, gender, educational level, current marital status, physical activities (type and frequency) and work (according to the National Economical Activities Classification). Participants will be asked to choose between the following physical activity statuses: sedentary, sedentary with light ambulation, light or vigorous physical activity.

**Clinical data** Weight and height, pain duration (in months), pharmacological treatment history (NSAIDs, analgesics, anxiolytic, skeletal muscle relaxants and corticosteroids (specifying type, duration and dosage)), physical therapy (type and duration), imaging and other radiological tests, neurophysiological tests, as well as work status will all be recorded. These data will be gathered by interviews and from the participants’ clinical history.

Comorbidity will also be considered. Different factors like hypertension, arthrosis, diabetes, heart disease, chronic obstructive pulmonary disease (COPD) or smoking can affect the course of low back pain and its disability. Comorbidity will be assessed with the Charlson comorbidity index (CCI) [49]. The CCI evaluates 13 different comorbid conditions. Each condition is assigned a score depending on the risk of dying associated with each one. Scores are added to provide a total score to predict mortality. Psychiatric comorbidity will be evaluated with the Brief Symptom Inventory (BSI) [50] adapted by Ruipérez et al. [51].

Finally, all the different interventions that patients could receive besides the treatments offered in this study will be recorded using the interview with the patient and the clinical history as sources. Pharmacological treatment, physical therapy (in public or private services) and infiltrations will also be recorded.

**Satisfaction with the treatment** The Spanish adaptation of the Borkovec and Nau questionnaire [52] will be used. Participants have to respond using an 11-point scale (0 to 10) to give their opinion on the treatment received (for example, how logical the treatment was for them; if they are satisfied; if the treatment was useful; and if they would recommend the treatment to other people). Clinicians will also answer an adapted version of the questionnaire regarding satisfaction with the treatment program.

### Experimental conditions

There are three experimental conditions: control group, CBT group and CBT with ICT group. All participants, regardless of group assignment, will receive the same rehabilitation treatment (back school), which will be carried out by the same physiotherapist blinded to group assignment. CBT sessions in the experimental groups will be administered by a psychologist who does not belong to the staff of the healthcare centre where the trial will take place (students of the PhD program or Master's degree at Universitat Jaume I and University of Valencia, without special clinical training). The psychologists of the project research team will conduct the training of the psychologists who will apply the CBT program.

### Control group (Rehabilitation treatment and information)

Patients will receive the traditional rehabilitation treatment (back school). This treatment will consist of a 4-session group therapy every week, with each session lasting 45 minutes. The content of the first session will be educational (ergonomics, pain demystification) and the other three will include physical therapy focused on stabilisation training: lower extremity stretching; finding the neutral spine position; spine stabiliser activation (transversus abdominis and multifidus); abdominal, spinal extensor and lower extremity strengthening; and proprioceptive control (stabilisation kinesitherapy) [53,54].

### CBT group (traditional cognitive behavioural therapy) (CBT)

Patients will receive the same treatment in physical therapy as the control group, in addition to CBT. The aim of the CBT intervention is to produce changes in the beliefs and behaviours about physical activity and avoidance of activity. The treatment’s components are:

– Psychoeducation to counteract the misconceptions about low back pain and to highlight the relevance of maintaining an adequate level of activity.

– Cognitive restructuring techniques to modify misbelieves about low back pain and reduce catastrophizing.

– Behavioural therapy and activity pacing to decrease avoidance behaviours linked to pain and promote physical activity and meaningful activities.

– Training on self-management pain techniques (mindfulness and relaxation).

It is a 6-session group therapy, with one session per week. Each group will be composed of 6 to 8 participants. Each patient in this group will have to attend the assessment sessions and at least 4 out of 6 CBT sessions.

### CBT + ICT group (cognitive behaviour therapy supported by ICT)

The patients of this group will receive the same interventions as the CBT group, but will receive a reinforcement of the sessions’ content through two different ways, both based on ICTs: a web tool named TEO (Emotional Therapy Online) specially designed to practice the therapeutic strategies at home; and SMS, which will be sent to the patient’s mobile phone with reminders and reinforcements. The software TEO has been designed by the team of psychologists on this project. It is a computer program that is accessed via the internet, aiming to allow participants to continue practicing at home what they have learned in the sessions. For this study, different sessions will be designed and developed. The content of the sessions will be related to the therapeutic components included in the CBT. Patients will access TEO from their homes using a personal password. Besides TEO, messages will be sent through SMS three times a week during the treatment and once a week during the follow-up. The messages will consist of reminders to do their homework, along with reinforcements of the effort made to improve their health.

### Analysis of the barriers for using CBT and ICT in chronic low back pain treatment

This analysis includes 3 groups of patients, resulting from the three experimental conditions, with 8 patients in each, selected by a balanced randomization to ensure variability in gender, age, educational level and the ability to use information and communication technologies. We will apply a focus group strategy that will be accomplished at the end of the therapy. The focus groups will be recorded and analyzed with qualitative analysis methodologies. The clinical staff’s opinion will be obtained using ad-hoc questionnaires.

### Collection and data analysis

Clinical and demographic data will be collected in the clinical interview and from the patient’s electronic health history. In the follow-up, we will collect the data of the questionnaires in the 3rd, 6th and 12th month. Due to the nature of the interventions, it will not be possible to blind the participants. Nevertheless, during the process, the assessors of the results will not know the group of the participants.

The first results will be obtained by intention-to-treat analysis. The time point of the primary objective analysis will be the 12th month, although the previous periods will also be analyzed.

Statistical descriptions will be obtained expressing the continuous variables in mean (standard deviation) or median (quartile) depending on normality or not, the categorical variable by number (percentage) and the 95% confidence interval for each case. Normal distribution will be checked with the Kolmogorov test to determine whether to use parametric or non-parametric tests. To compare continuous variables among the groups, Student’s T-test and Mann-Whitney’s U-test will be used, and for categorical variables, Chi-square or Fisher test, depending on the kind of distribution.

Intra-group changes in the continuous variables along the time points will be explored with Student’s T-test for paired samples. To assess if there are differences among the groups according to pain, disability and the rest of the variables, we will use analysis of covariance (ANCOVA) for each dependent variable. In all the tests, the independent variable will be the group, while the covariate variable will be the basal level of each dependent variable. It will be necessary to have comparable levels of both types of variables to understand correctly the ANCOVA results. Therefore, we will perform different Student’s T test estimations, one for each dependent variable, considering each group as an independent variable in each case. To evaluate whether the changes in the three groups have happened in a different way, we will estimate different ANOVA, an analysis for each dependent variable. In all cases, the independent variable will be the group and time in which the data are collected. In this analysis, we will also study possible interactions. Furthermore, we will evaluate the correlations between the variables using Rho-Pearson and Spearman according to normality. We will also use two multiple linear regression models for pain and disability as dependent variables and in both models, gender, age, pain, catastrophizing, fear avoidance, days taken off at work and time evolution will be employed as independent variables.

### Privacy and data protection

Personal passwords and data encryption as Advanced Encryption Standard (AES) will be used. The project will comply with current guidelines in Spain and EU for patient protection in clinical trials according to collection, storage and the keeping of personal data.

### Ethical issues

The trial will comply with Helsinki’s Declaration. This project has been approved by the ethics research committee of Hospital Arnau de Vilanova in Valencia. As for the intervention and assessment protocols used in this study, there is nothing that poses a risk to the participants, according to existing knowledge. The treatment protocols to be developed are based on CBT, which is first and foremost a learning- or teaching-based therapy. Thus, the treatment is not invasive at a cognitive level, except as far as any learning or teaching is concerned.

## Discussion

There is no gold standard therapy for chronic low back pain. Thus, it is necessary to find new treatment strategies that improve on current results. In this work, we propose to apply a randomized controlled clinical trial to test the effectiveness of a CBT intervention using ICT or not for treating CLBP. The ICT will use the online program TEO and mobile phone SMS. TEO is a completely open system that allows therapists to create and show personalized therapeutic material to patients online. The primary data concerning TEO system’s acceptance by patients has already been obtained [55].

Internet use in the Spanish population is high and to rise, with Spain taking the 7th position in Europe with regard to Internet use. In 2012, two out of three homes had broadband connection, 8.0% more than in 2011 [56]. Therefore, the internet is a promising way of delivering information and interacting with patients. If the therapy designed in this project is effective, it could be applied in daily clinical practice to improve CLBP treatment.

Moreover, this trial opens the door to new research that could explore the possibility of using certain therapies, or some of them, in the patient’s own home. In this way, the healthcare burden of treating CLBP at a clinical setting could be reduced. Moreover, treatment adherence can be improved.

These results will support and contribute to the literature, indicating the great potential for technological adjuncts like the ones designed in the present study. Engagement during and between therapy sessions could be enhanced by employing electronic homework and aftercare practices to increase adherence and patient satisfaction [57].

If the results of this study are positive, there will be a manualized CBT program that could be used easily in the daily care of CLBP patients and applied in two versions: traditional (for those patients without internet access) and with the support of internet and SMS. It will be possible to train therapists easily on administering this program, not requiring specialised training. This treatment program will contribute to improving the resources included in the clinical guidelines for CLBP treatment.

This study has some limitations. Although the randomized sequence will be hidden, due to the nature of the treatments, it will not be possible to blind the participants to the group that has been assigned to them. It is possible that patients in the CBT groups will feel more looked after, therefore giving more pleasing answers. Despite not being a blinded placebo, the control group will also attend the back school group sessions to try to minimize this effect. We have assumed that patients in the three groups will have the same treatment apart from the interventions included in the study. During the trial, it may be possible that the blinding of the clinicians and physiotherapists is lost, leading to possible selective co-interventions. Patients will be asked not to tell their clinicians, physiotherapists or orthopaedic surgeons their treatment group. Their clinical history will only note that the patient attends educational sessions for CLBP.

Most of the clinical trials that have studied CBT modalities in other countries have been performed with patients in primary care. This project will be carried out in specialized care in the Spanish healthcare system, which reaches all the population and whose access is controlled by primary care. The results of clinical trials on CBT tend to be much better in specialized care than primary one, possibly because of a selection bias that causes the patients in specialized care to be usually in a worse condition [58]. Patients will continue to be treated by their primary care doctors to whom they have free access. The recommendations that doctors may give can vary due to their own beliefs and attitudes about back pain, regardless of their knowledge about guidelines [59].

We hope this work will offer useful data about the short- and long-term efficacy of a treatment of disability and pain. This could open new avenues for developing public health strategies and treatments, leading to a better care of the patients suffering from this problem.

## Competing interests

The authors declare that they have no competing interests. The study sponsors have no role in the study design, the collection, analysis, or interpretation of the data, the writing of the report, or the decision to submit the paper for publication.

## Authors’ contributions

JD in collaboration with RB and CB designed the study. JD and AGP drafted the manuscript. RB, CB and RH designed the cognitive behavioural intervention and elaborated the educational online software. LP and AE contributed to the design and description of the physical therapy treatment CB, RB, RH, LP, AE, MM, JH and JB reviewed and revised the manuscript, making significant improvements. All authors have read and approved the final manuscript to be published.

## Pre-publication history

The pre-publication history for this paper can be accessed here:

http://www.biomedcentral.com/1471-2474/14/142/prepub
